# A Conserved GPG-Motif in the HIV-1 Nef Core Is Required for Principal Nef-Activities

**DOI:** 10.1371/journal.pone.0145239

**Published:** 2015-12-23

**Authors:** Marta Martínez-Bonet, Claudia Palladino, Veronica Briz, Jochen M. Rudolph, Oliver T. Fackler, Miguel Relloso, Maria Angeles Muñoz-Fernandez, Ricardo Madrid

**Affiliations:** 1 Laboratorio de Inmunobiología Molecular, Instituto de Investigación Biomédica Gregorio Marañón (IISGM), 28007 Madrid, Spain; 2 Department of Infectious Diseases, Integrative Virology, University Hospital Heidelberg, Heidelberg, Germany; 3 Departament of Virology. Centro de Biología Molecular Severo Ochoa, CSIC/UAM, Madrid, Spain; George Mason University, UNITED STATES

## Abstract

To find out new determinants required for Nef activity we performed a functional alanine scanning analysis along a discrete but highly conserved region at the core of HIV-1 Nef. We identified the GPG-motif, located at the 121–137 region of HIV-1 NL4.3 Nef, as a novel protein signature strictly required for the p56^Lck^ dependent Nef-induced CD4-downregulation in T-cells. Since the Nef-GPG motif was dispensable for CD4-downregulation in HeLa-CD4 cells, Nef/AP-1 interaction and Nef-dependent effects on Tf-R trafficking, the observed effects on CD4 downregulation cannot be attributed to structure constraints or to alterations on general protein trafficking. Besides, we found that the GPG-motif was also required for Nef-dependent inhibition of ring actin re-organization upon TCR triggering and MHCI downregulation, suggesting that the GPG-motif could actively cooperate with the Nef PxxP motif for these HIV-1 Nef-related effects. Finally, we observed that the Nef-GPG motif was required for optimal infectivity of those viruses produced in T-cells. According to these findings, we propose the conserved GPG-motif in HIV-1 Nef as functional region required for HIV-1 infectivity and therefore with a potential interest for the interference of Nef activity during HIV-1 infection.

## Introduction

Nef is a myristoylated accessory protein of about 25- to 34 kDa produced early and abundantly upon infection by HIV-1, HIV-2 or SIV. Nef is a molecular regulator of HIV-1 infectivity and pathogenesis and its expression increases virus titers by more than two logs during the early phase of infection [1,2]. This feature likely favours initial viral spread and the onset of AIDS in infected patients [3,4,5,6]. Nef is a multifunctional viral protein designed to interact with different host factors. Among the different interaction sites described so far, Nef protein includes a Proline-rich domain (_72_PxxP_75_) that allows interaction with the SH3 domain of the Src family tyrosine kinase proteins (as Hck, Lyn and lymphocyte-specific protein tyrosine kinase p56^Lck^); the acidic motif (_61_EEEE_64_) that mediates the interaction with PACS-1 protein, and the di-Leucine domain (_160_ExxxLL_165_), involved in the interaction with the clathrin adaptor complexes AP-1, AP-2 and AP-3 [7]. Mainly through direct protein-protein interactions, the Nef’s hallmark lies on its ability to fine-tune the intracellular transport and signal transduction processes to optimize viral replication and spread ([8,9] for review). Additionally, HIV-1 Nef contributes to the escape of HIV-1-infected cells from immunosurveillance by altering the subcellular localization of several key proteins in the immune response such as CD4 [10], MHCI [11], CD28 [12], CD8b [13], CXCR4 [14], CCR5 [15], MHCII [16], and DC-SIGN [17], mainly through the recruitment of the ubiquitous adaptors complexes AP-1 and AP-3 [18,19,20,21], impairing the endocytic and the anterograde trafficking routes in infected cells [22,23].

Consider the Nef-induced CD4-downregulation from the plasma membrane. Although Nef is able to inhibit its recycling and promote its recruitment into late endosomes towards protein degradation in all the cell types tested so far, HIV-1 Nef is further able to boost the internalization rate of CD4 but only in lymphoid cells [24]. This early effect has been broadly accepted to occur through the impairment of p56^Lck^-mediated CD4-membrane stabilization by Nef [25]. Indeed, it has been demonstrated that Nef should first disrupt the p56^Lck^/CD4 interaction to unmask the CD4 di-Leucine motif and subsequently recruits AP-2 to discrete regions of the plasma membrane to enhance CD4 internalization rates [24,25,26].

Besides its role in CD4 stabilization at plasma membrane, p56^Lck^ is also central for HIV-1 Nef activity at tuning the activation levels in infected CD4+ T cells and extending their life-span during viral replication. In particular, HIV-1 Nef affects the phosphorylation status of p56^Lck^, its activity and induces its redistribution into an intracellular compartment (IC) located at the Recycling Endosomes/Trans-Golgi Network (RE/TGN) level [25,27,28,29]. This effect on p56^Lck^ is a well conserved feature along Nef proteins [30]. At molecular level, the integrity of the Nef-PxxP domain is required for the related effects of HIV-1 Nef on p56^Lck^. Studies on Nef/p56^Lck^ interaction indicate that the Nef-PxxP domain is required but not sufficient to allow the binding to p56^Lck^, since the interaction with the p56^Lck^ -SH2 domain synergistically enhances the binding of HIV-1 Nef and p56^Lck^ [25,31]. The PxxP motif is also essential for downregulation of MHCI [32], as well as for the association of Nef with the cellular kinase PAK2, which causes the phosphorylation and thereby inactivation of the actin severing factor cofilin, inducing cytoskeletal rearrangements and thus preventing the formation of F-actin rich circumferential rings [33,34]

In this study, we identified a novel functional motif in a highly conserved loop in Nef proteins, the GPG-motif, required for the main activities of HIV-1 Nef, such as CD4-downregulation, p56^Lck^ recruitment and optimal infectivity of viruses produced in T-lymphocytes, and involved in ring actin re-organization inhibition and MHCI downregulation.

Therefore, we propose the Nef-GPG motif as target for therapeutic intervention to restrict key Nef activities during the early phases of infection and dissemination of HIV-1.

## Materials and Methods

### Plasmid constructs

pCG-Nef-GFP, pGEX-Nef or pCDNA-Nef-HA for the expression of Nef-GFP, GST-Nef and Nef-HA constructs, respectively, were generously donated by S. Benichou (Institut Cochin, Paris, France) and used as templates for paired mutagenesis (Quick-change site directed mutagenesis Stratagene, La Jolla, CA) in the 123–137 region of HIV-1 Nef using specific mutagenic primers (S1 Table). Correctness of mutations was verified by automated DNA sequencing and blast analysis. X4-tropic strain pNL4.3 (HIV-1 wt) and pNL4.3 Δnef (HIV Δnef) (NIH; NIH AIDS Research and Reference Reagent Program, Division of AIDS, NIAID). p56^Lck^-GFP and p56^Lck^ -mCherry constructs were obtained by the fusion of full-length human p56^Lck^ cDNA in pEGFP-N1or pmCherry N1 (Clontech), respectively.

### Antibodies

Mouse antibodies for γ-adaptin and GFP were obtained from Sigma; rat anti-HA clone 3E10 was from Roche; anti-human CD4 mAbs clone 4B12 and clone OKT4 were from Santacruz and Leica; purified anti-MCHI mAb (WG-32) was kindly donated by A. Corbí (CIB-CSIC, Madrid); anti-Tf-R mAb was from Zymed and Alexa-594 Transferrin conjugate was from Molecular Probes. Anti-p56^Lck^ mAb was from BD Transduction Laboratories and pTyr505-Lck mAb was from Millipore. PE-, PC7-conjugated anti-human CD4 mAbs, PE-conjugated anti-CD3 mAb, and PE- and PC7-conjugated anti-mouse immunoglobulin G were purchased by BD Pharmingen. Secondary antibodies Alexa-555- or Alexa-647- conjugated anti-murine, and Alexa-555-conjugated anti-rat were from Molecular Probes. For T cell spreading, the monoclonal antibody (mAb) anti-CD3 (clone HIT3a) was used (BD Pharmingen). For F-actin stain, tetramethylrhodamine isothiocyanate (TRITC)-phalloidin (Sigma) was used. For Western blotting, secondary horseradish peroxidase-conjugated anti-IgGs were from Dako.

### Cells and transfections

HeLa P4R5 MAGI, HeLa TZM-bl (NIH; NIH AIDS Research and Reference Reagent Program, Division of AIDS, NIAID) and HEK 293T cells (ATCC number CRL-11268, Rockefeller University) were grown in DMEM plus glutamine, antibiotics and 5% decomplemented-FCS (foetal calf serum) (GibcoBRL, Invitrogen). HeLa P4R5 MAGI cell cultures were supplemented with 100 mg/mL geneticin and 1 mg/mL puromycin. T-cell lines were maintained in RPMI with 10% FCS and supplemental glutamine, penicillin, and streptomycin. For experiments with human primary T cells, the CD3+CD4+ T cell subpopulation was obtained by negative selection (Miltenyi) from freshly PBMC from healthy donors and immediately activated for 3 days with 1 μg/mL PHA, 30 U/mL IL2 in RPMI with 10% fetal bovine serum and supplemented with glutamine, penicillin, and streptomycin. CEM-SS and Jurkat cells (NIH; NIH AIDS Research and Reference Reagent Program, Division of AIDS, NIAID) as well as isolated primary T-lymphocytes were transfected by electroporation using the Nucleofector I device (Lonza) according to manufacturer's guidelines. HeLa and HEK-293T cells were transfected using Lipofectin (Invitrogen).

### Flow cytometry analysis of CD4 downregulation

CEM-SS cells, HeLa P4R5 MAGI or primary T-cells were transfected with 20 μg of plasmids expressing wild-type or mutated Nef-GFP plasmids. When specified, HeLa P4R5 MAGI cells were cotransfected with 5 μg of p56^Lck^-GFP plasmid and 10 μg of plasmids expressing wild type or mutated Nef-HA. After 16 h, cells were stained for 1h at 4°C with the corresponding conjugated antibodies in FACS staining buffer (phosphate-buffered saline, PBS) with 2% fetal bovine serum and IgGs from murine serum (SIGMA). Specific staining in CEM-SS and HeLa P4R5 MAGI cells was analysed by two-colour flow cytometry, whereas in primary T-cells three-colour flow cytometry was carried out in a FACSCalibur flow cytometer (Becton Dickinson). FlowJo software (FLOWJO LLC. http://www.flowjo.com/) was used to quantify the mean fluorescence intensity (MFI) of non.transfected and transfected cells. The mean fluorescence intensity (MFI) of the GFP-positive cells was used to calculate the % of activity of each mutant in reducing the surface expression of CD4 relative to wild-type Nef and expressed as the mean ± SD of three different assays done in duplicate.

### Flow cytometry analysis of MHCI downregulation

CEM-SS cells were transfected with 15 μg of plasmids expressing wild-type or mutated Nef-GFP plasmids. After 24 h, the cells were first stained for 1h at 4°C with an anti-MHCI antibody and then stained with a secondary PE-conjugated antibody in FACS staining buffer. Specific staining was analysed by two-colour flow cytometry using a FACSCalibur flow cytometer (Becton Dickinson). FlowJo data analysis software (FLOWJO. LLC. http://www.flowjo.com/) was used to quantify the mean fluorescence intensity (MFI) of nontransfected and transfected cells. The MFI of the GFP-positive cells was used to calculate the % of activity of each mutant in reducing the surface expression of MCHI relative to wild-type Nef and expressed as the mean ± SD of three different assays done in duplicate.

### Indirect immunofluorescence confocal microscopic analysis

CEM-SS, Jurkat, primary T-cells or HeLa P4R5 MAGI cells spread on poly-L-lysine (Sigma) and normal glass coverslips, respectively, were stained for immunofluorescent analysis as described [22]. Cells were fixed in formalin (Sigma), quenched for 5 min with 0,1% (wt/vol) BSA in PBS, and permeabilized or not with 0.1% Triton-X100 (Sigma) in PBS at 4°C for 5 min, rinsed, incubated with 1% (wt/vol) BSA (Sigma) for 30 min. Cells were then incubated successively with the primary and the appropriate fluorescent secondary antibody for 1h at room temperature. For double-labelling experiments, a sequential immunostaining procedure was followed. Cell surface staining of markers was carried out at 4°C in non permeabilized cells prior to fixation. Suitable controls to assess the specificity of the labelling were performed. After thorough washing, coverslips were mounted on slides using Fluoromount-G (SouthernBiotech). Images were acquired using a confocal laser microscope (Leica TCS SP5, Leica Mycrosystems). Fluorescence acquisition was performed using the 488-, 561- or 647-nm laser lines. For deconvolution Huygens 3.0 software was used and images were processed using Adobe Photoshop software. For each sample, three medial optical 0.5 μm slices of 50 different cells were recorded. Image J software (National Institutes of Health; (http://rsb.info.nih.gov/ij/index.html) was used to quantify the intensity of fluorescence (mean intensity of fluorescence per pixel) of CD4, p56^Lck^, pY505-Lck, Tf-R distribution at the plasma membrane or endosomal compartments in the three medial confocal sections using a 0–250 gray color scale. For the quantitative analysis of p56^Lck^ distribution in Jurkat and PBLs, the p56^Lck^ fluorescence signal plotted along 8 radial lines at the equatorial plane of the cell using the Image J and the delimited surfaces were measured. The ratio of internal to plasma membrane fluorescence of p56^Lck^ was calculated for each mutant in three independent experiments (n = 25 cells). Cells with a ratio greater than or equal to the mean number of non-transfected cells+2×SEM were considered to express high levels of p56^Lck^ at intracellular compartments. Only 8% of non-transfected cells met this standard. The confidence limits of the results were assessed by Student’s *t* test.

### Transferrin uptake assays

16h after transfection, CEM cells were attached to poly-L-lysine glass coverslips and starved in serum-free medium (containing 0.1% BSA, 10 mM HEPES) for 2 h at 37°C. Cells were then incubated at 37°C for 20 min in the same medium containing 10 μg/ml Tfn-Alexa-594. Cells were washed, fixed, and then analysed by confocal microscopy (Leica TCS SP5, Leica Mycrosystems). Double fluorescence acquisition was performed using the 488- and 561-nm laser lines. In each experiment, the laser beam and the photomultipliers were adjusted to the Alexa signal of non-transfected cells. For each sample, three medial optical 0.5 μm slices of 50 different cells were recorded. NIH Image software was used to quantify the intensity of fluorescence (mean intensity of fluorescence per pixel) of Tfn-594 staining on the whole cells using a 0–250 grayscale. The confidence limits of the results were assessed by Student’s *t* test. For deconvolution we used Huygens 3.0 software was used and images were processed using Adobe Photoshop software.

### Analysis of TCR-mediated actin ring formation

T-cell spreading on stimulatory surfaces was performed essentially as described [35]. Briefly, microscope cover glasses were coated with anti-CD3 antibody diluted in TBS (10 μg/ml) for 3h at 37°C. After washing with TBS, the cover glasses were stored in TBS at 4°C. Cells were added in a volume of 50 μl onto the glasses, incubated for 5 min at 37°C and fixed by direct addition of 4% PFA. Cell were then permeabilized as described above and incubated with TRITC-phalloidin for 5 min at RT. Further steps were performed as described above.

### GST pull-down assay

Full length Nef protein fused to glutathione-*S*-transferase (GST) was produced in DH5 α *E*.*coli* cells and purified by affinity using GSH-Sepharose beads (Amersham Biosciences). For Nef/AP-1 binding, HeLa cells (5.10^6^) were lysed in Lysis buffer (25 mM Tris-HCl pH 7.4, 2 mM EDTA, 120 mM NaCl, 1% glycerol, 0,5% Triton and protease inhibitors). The cleared lysates were incubated for 2h or overnight at 4°C with 3 μg of GST or GST-Nef proteins immobilized on glutathione (GSH)-Sepharose beads. Then, beads were washed three times in lysis buffer, and the bound cellular proteins were analysed by Western blotting using the appropriate antibodies. The chemiluminescent signals were quantified using Image J.

### Viral infectivity

Virions were transduced by transient transfection of Jurkat (6.10^6^) or HEK 293T cells (5.10^6^) with 20 μg of proviral pNL43-wt, or 20 μgof proviral pNL43- Δnef plasmid along 60 μg of empty PCI or expression plasmids for wild-type or mutated Nef-HA. 72h post-transfection, virion-containing culture supernatants were harvested, pre-cleared by centrifugation at 1,200 rpm for 7 min and filtered through a 0.45-μm-pore-size membrane. The concentration of p24 antigen in viral stocks was measured by a quantitative enzyme-linked immunosorbent assay, ELISA (Immunogenetics), prior infectivity assays in TZM-bl cells. TZM-bl cells were infected as already described [36]. Briefly, cells were seeded one day before, infected with 20 ng p24/10^6^ cells, and incubated for 3h at 37°C. Then, cells were incubated to 37°C upon removal of unbound virus by extensive washes. 48h after infection, TZM-bl cells were lysed using Glo-Lysis buffer (Promega) and luciferase assays were performed according to manufacture recommendations (Promega). Luminescence was measured in a VICTOR luminometer. Results were normalized to a value of 100% infectivity and represented as mean ± SEM of relative luciferase units.

### Analysis of 3D structure

Structural 3D models were built using Pymol software (Schrödinger, LLC) from the PDB file DOI:10.2210/pdb2nef/pdb at http://www.rcsb.org/pdb/home/home.do

### Western blot analysis

Expression of wild-type and mutated Nef-GFP, p56^Lck^-GFP, Nef-HA, endogenous p56^Lck^, p56^Lck^-Cherry, or **γ**-adaptin, from transfected cells was analysed by Western blotting using anti-GFP, HA, anti-p56^Lck^ and anti-**γ**-adaptin primary antibodies. Image J was used to quantify the blots.

### Ethics statement

Freshly isolated T lymphocytes from healthy donors were used following the guidelines of the Bioethics Committee of the Spanish Research Council and the institutional management committee of the Centro de Biología Molecular “Severo Ochoa”. Human peripheral blood mononuclear cells (PBMC) were isolated from buffy coats of healthy informed subjects provided by the Madrid Transfusion Centre (http://www.madrid.org/cs/Satellite?language=es&pagename=CentrodeTransfusion%2FPage%2FHLAV_home) following national guidelines.

### Statistics

p values indicate statistical significance as determined by Student’s t test (*, p< 0.05; **, p< 0.01; ***, p< 0.001).

## Results

### Identification of the GPG-motif in HIV-1 Nef

**Fig 1:**
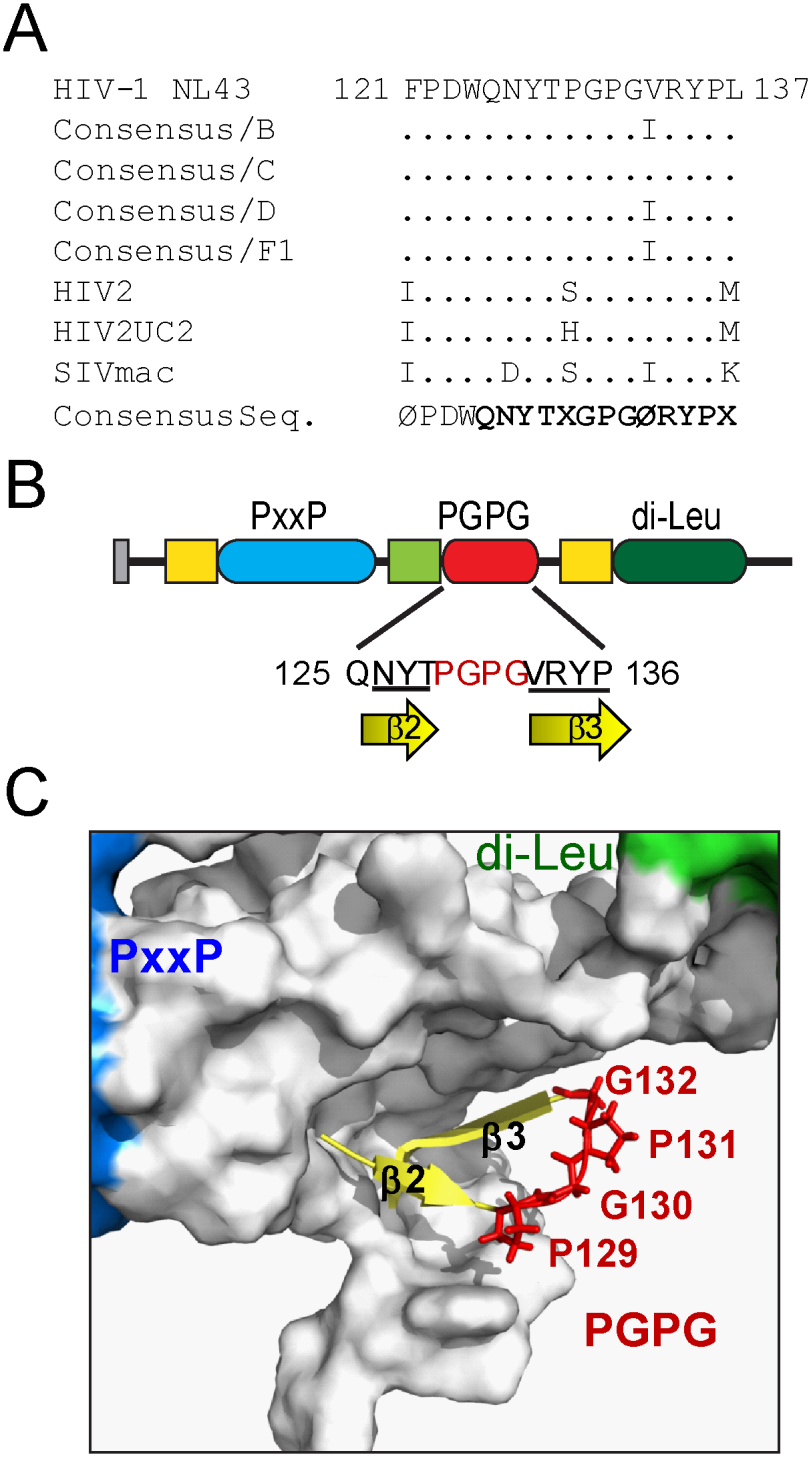
The GPG-loop is conserved among Nef proteins. A) Clustal W alignment of the 121–137 region of HIV1- Nef with homologous HIV2- and SIV-Nef sequences from Los Alamos database. The consensus sequence of the GPG-loop is highlighted to indicate the amino acid identity. X represents any amino acid residue and Ø represents a hydrophobic residue. B) Schematic representation of the domain organization of HIV-1NL4.3 Nef. The novel GPG-loop (red), conserved PxxP domain (blue), the di-Leucine based motif (dark green) as well as the membrane anchorage domain (grey), the acidic clusters (orange) and the dimerization domain (light green) are shown. C) Ribbon/surface diagram showing the HIV-1 Nef-GPG loop as generated by using the Pymol software (pdb2nef). The central GPG residues (red) are flanked by hydrophobic β2 and β3 sheets (yellow) in HIV-1 Nef structure. PxxP and di-Leucine motifs surfaces are coloured in blue or green, respectively.

We piled-up the HIV-1, HIV-2 and SIV Nef protein sequences available at the Los Alamos HIV database (consulted in January 2015) to unveil new determinants involved in HIV-1 Nef’s effects, using the Clustal Omega software (http://www.ebi.ac.uk/Tools/msa/clustalo/). Among the overall similarities found we focused on the conserved region 125 to 136 of HIV-1 NL4.3 Nef, represented by the consensus sequence QNYTXGPGØRYP (Fig 1A and 1B).

This uncharacterized region forms a small loop between the two anti-parallel β-strands (β2: NYT and β3: VRYP) that expose the hydrophobic PGPG residues at the surface of Nef (Fig 1C). Consequently and because of its conservation along the different Nef alleles, we will hereafter refer to this region as the GPG-loop. This loop is located immediately downstream of the dimerization (_121_FPD_123_), not close to the SH3-binding Proline-rich (_69_PxxP_75_) or to the di-Leucine motif (_160_ENTSLL_165_).

### Mutations on the Nef-GPG loop abrogate Nef-induced CD4 downregulation in T-cells

We first explored the potential requisite of the GPG-loop for HIV-1 Nef’s effects on CD4 downregulation. CEM T-cells were transfected with expression constructs coding the different Nef-GPG mutants (Table 1) and screened for CD4 surface expression levels.

**Table 1:** Amino acid sequences of wild-type and mutated HIV-1 Nef NL4.3 from Phe121 to Phe137.

HIV-1 Nef	121	FPDWQNYTPGPGVRYPL	137
D123A		--**A**--------------	
DW/AA		--**AA**-------------	
QN/AA		----**AA**-----------	
YT/AA		------**AA**---------	
PG1/AA		--------**AA**-------	
PG2/AA		----------**AA**-----	
VR/AA		------------**AA**---	
YP/AA		--------------**AA**-	

**Fig 2:**
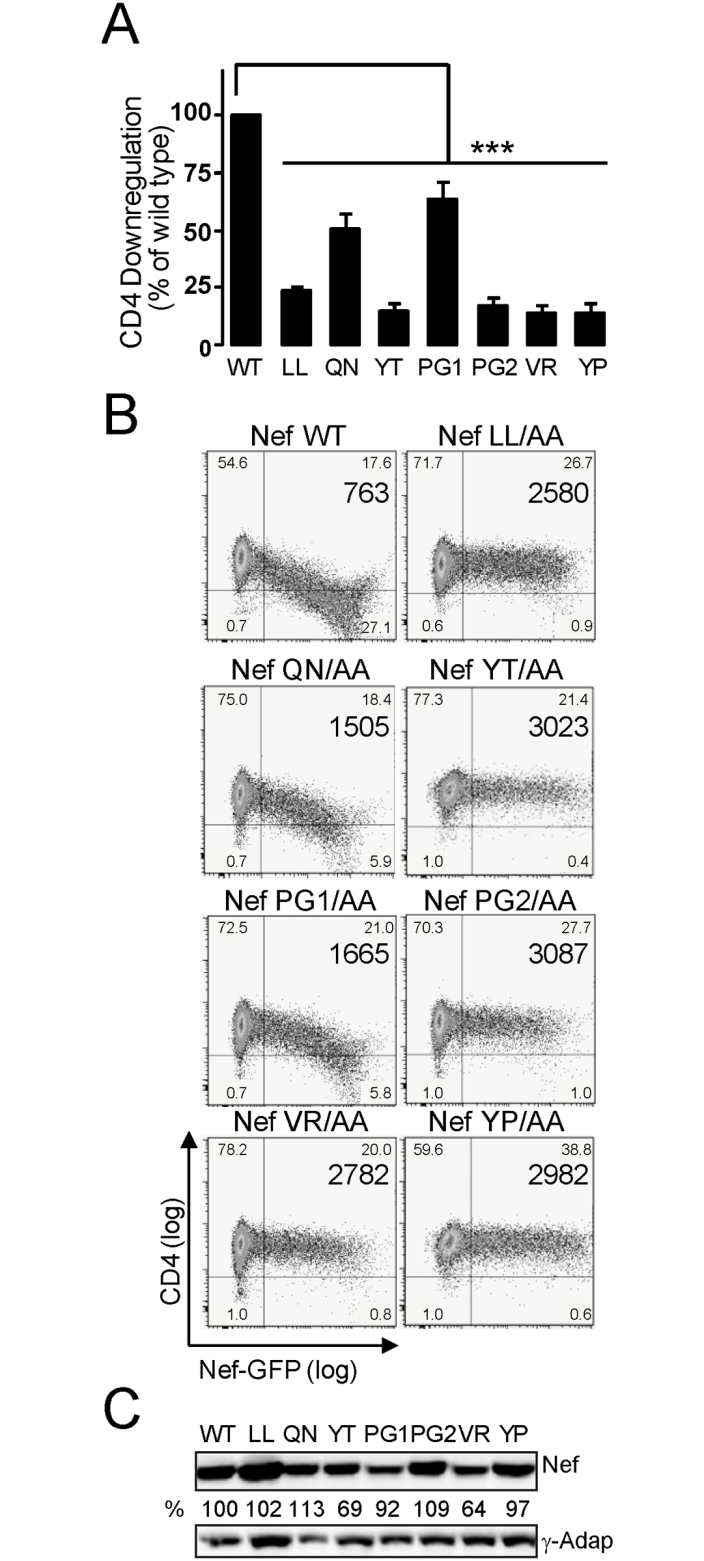
Mutations on the GPG-loop abrogate HIV-1 Nef-induced CD4 down-regulation. A-B) CEM cells were transfected with either wild type or the indicated Nef mutants as GFP-fusion proteins. Nef-induced surface CD4 down-regulation was analysed by two-colour flow cytometry in Nef-GFP expressing cells. The mean CD4 (PE) fluorescence intensity (MFI) of the GFP-positive cells was used to calculate the Nef activity on surface CD4 expression (A). Histograms represent the percentage of activity relative to wild-type Nef as the arithmetic mean ± SD of at least three independent experiments. Dot plots and the corresponding MFI are representative of three independent assays (B). ***, p< 0.001. C) The expression of wild type and the indicated Nef-GPG mutants in CEM cells was analysed by western blotting using an anti-GFP antibody. **γ**-adaptin levels were used as loading control staining.

Flow cytometry analysis showed that Nef WT induced a robust reduction of CD4 cell surface levels (Fig 2A and 2B). In contrast, the YT/AA, PG2/AA, VR/AA or YP/AA mutations in the GPG-loop induced a 5–6 fold reduction in Nef-mediated CD4-downregulation, displaying surface CD4 levels close to cells expressing either the Nef-LL/AA deficient mutant (Fig 2A and 2B) or the dimerization mutants (S1A and S1B Fig).

CD4 downregulation by the Nef-QN/AA or -PG1/AA mutants was also significantly impaired but just showed a 2-fold reduction compared to wild type Nef. The reported effects of GPG mutants were not related to differences on Nef expression levels (Fig 2C), and were further confirmed in CD3+CD4+ T-cells (S1C and S1D Fig).

### GPG-dependent CD4 downregulation by HIV-1 Nef is strictly related to p56^Lck^ expression

**Fig 3:**
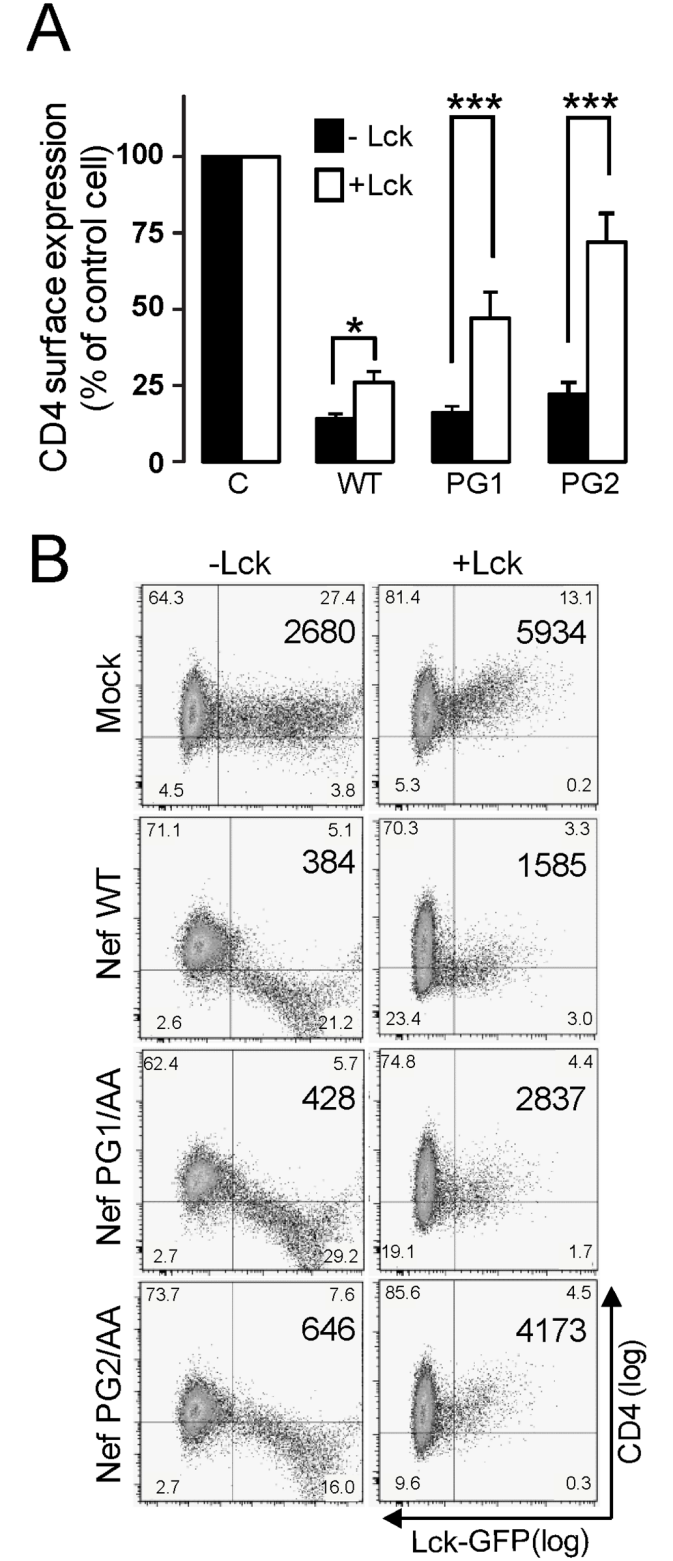
The integrity of the GPG-motif is dispensable for Nef-induced CD4-downregulation in p56^Lck^ -negative cells. A) HeLa-CD4 cells were co-transfected with the wild type or the indicated Nef-GPG mutants (Nef-HA) along with empty or p56^Lck^-GFP constructs. Nef activity on CD4-downregulation was analysed by two-colour flow cytometry in GFP cells. A) Histograms represent the percentage of CD4 expression at cell surface relative to non-transfected cells. n.s, not statistically significant. ***, p< 0.001. B) Dot plots and the corresponding MFI are representative of three independent assays.

We next analysed the effects of GPG mutations on Nef-induced CD4-downregulation in p56^Lck^ -negative cells. HeLa-CD4 cells were co-transfected with the mutated Nef-GPG expression plasmids along empty or p56^Lck^ expressing vectors. Surface and total expression of CD4 was analysed by FACS (Fig 3) or immunofluorescence (S2 Fig), respectively.

Both approaches showed that the central residues of the GPG-loop were dispensable for Nef activity on CD4-downregulation in HeLa-CD4 cells. Nef WT induced a robust reduction of CD4 cell surface levels, and this effect was not significantly affected by the expression of either Nef-PG1/AA, -PG2/AA (Fig 3A), or -QN/AA (S2 Fig) compared to wild type Nef. In contrast, the Nef-YT/AA, -VR/AA and -YP/AA mutants (S2A Fig) were as defective as in T-cells. Of note, in HeLa-CD4/p56^Lck^ cells, the central Nef-GPG mutants recovered its defective phenotype since CD4-downregulation was as impaired as in T-cells (Fig 3B and S2B Fig). The reported effects of GPG mutants were not related to differences on Nef expression levels (S2C and S2D Fig).

Since the central GPG residues were required in cells expressing p56^Lck^ but dispensable for CD4-downregulation in HeLa-CD4 cells, these results indicate that their mutation does not seem to impair Nef function through structural modifications. Moreover, our data point out that the GPG-motif is a functional signature in HIV-1 Nef.

### Nef-GPG motif is not involved in Nef-induced effects in endosomal trafficking

Besides CD4, HIV-1 Nef causes a general effect on membrane trafficking within the endocytic pathway in host cells that affects the expression of transferrin receptor (Tf-R) at plasma membrane [22,23,24]. Indeed, HIV-1 Nef slows down the recycling rate of Tf-R and promotes its intracellular retention without affecting its Transferrin (Tfn)-dependent internalization rate [22]. This effect has been demonstrated to be dependent on the integrity of Nef di-Leucine motif through a direct binding to and the recruitment of AP-1 and AP-3 complexes at the early/recycling endosomal compartment level [20,22].

Thus to further exclude a negative effect on key motifs required for CD4-downregulation, we analysed whether the mutations on the GPG-motif affected either the interaction of Nef with the AP-1 clathrin complex and/or the Nef-induced impairment of endosomal traffic of Tf-R.

**Fig 4:**
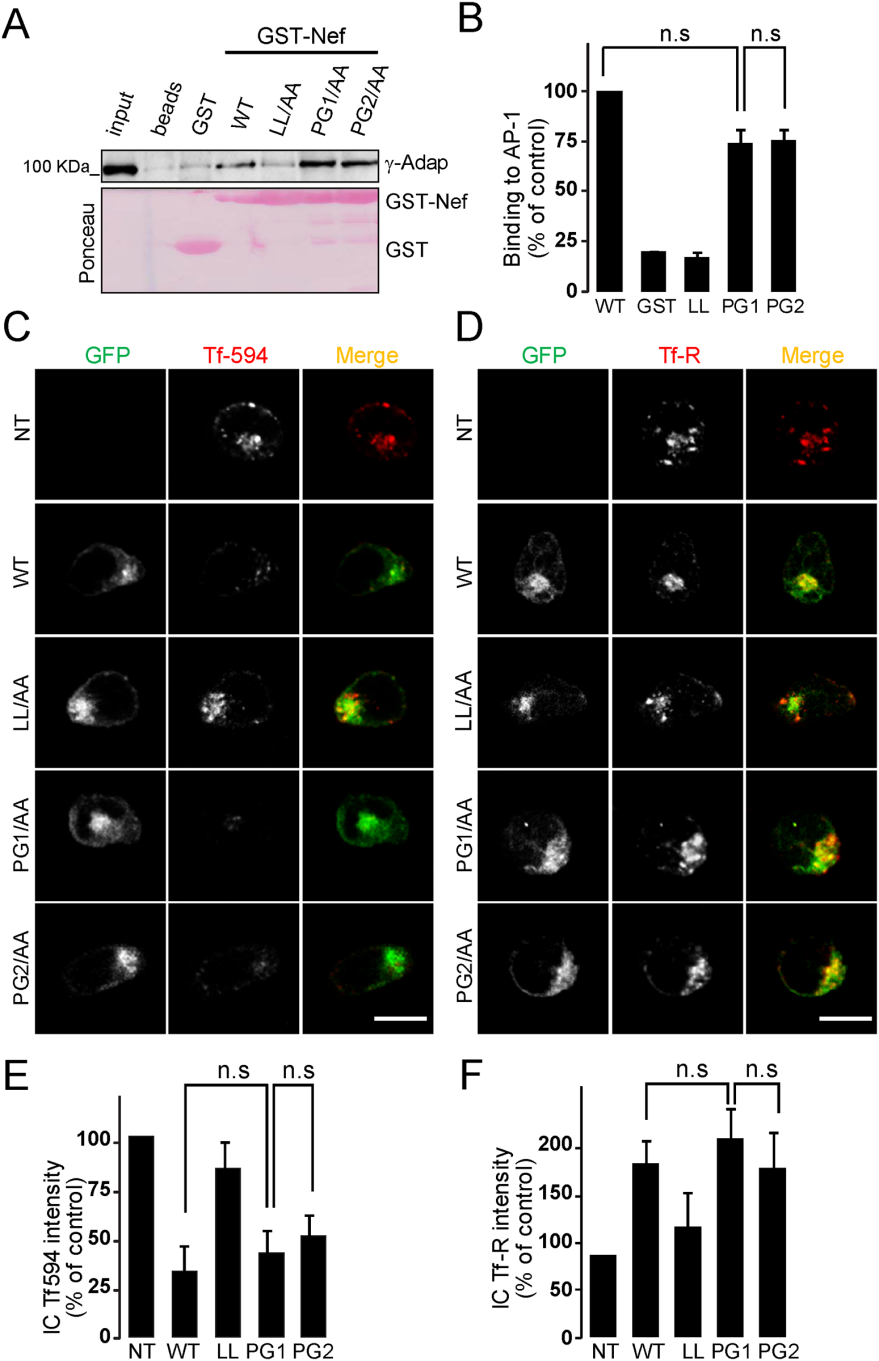
GPG mutations do not affect Nef-induced alteration of Tf-R trafficking. (A-B) Analysis of Nef/AP-1interaction by i*n vitro* pull-down. HeLa cell lysates were incubated with equal amounts of GSH-sepharose beads, or purified GST, wild type or the indicated Nef mutants as GST-fusions previously immobilized on GSH-sepharose beads. Bound proteins were resolved by SDS-PAGE and the association of AP-1 complex was analysed by Western blotting with anti-**γ**-adaptin (A) and quantified by densitometry (B). Ponceau-red staining shows the loading of the assayed Nef-GST fusion proteins on the beads. n.s not statistically significant. (C-D) Non-transfected, NT, or transfected CEM-T cells with expression plasmids for wild type or the indicated Nef-GPG mutants as GFP-fusion proteins were serum-starved and then incubated with Tfn-Alexa-594 for 15 min at 37°C before fixation (C), or analysed by indirect immunofluorescent staining for total Tf-R in permeabilized cells (D). Staining was visualized by confocal microscopy. A medial optical section of a representative cell is shown. Merged images show both GFP and Tfn-Alexa-594 or Tf-R labelling. Scale bar, 10 μm. (E-F) The intensity of the fluorescence corresponding to Tfn-594 (E) or Tf-R (F) was quantified by densitometry as described under Materials and Methods. Histograms represent the percentages of the mean fluorescence intensity relative to non-transfected cells (lower panel). Values are the arithmetic mean ± SD of at least three independent experiments in which over 50 cells were counted per condition. n.s. not statistically significant.

The Nef-PG1 and -PG2 mutants did not seem to negatively affect the Nef/AP-1 interaction (Fig 4A and 4B), the Nef-induced Tf-R downregulation from cell surface (Fig 4C and 4E), or its intracellular recruitment (Fig 4D and 4F) compared to wild type Nef.

Therefore, our results indicate that the observed GPG-dependent effects on CD4-downregulation are not related to the integrity of the Nef di-Leucine motif. Altogether, our results suggest that the central GPG residues in Nef are not a structural but a functional motif that operates independently of the Nef di-Leucine motif to enable CD4 downregulation.

### Role of the GPG-motif in Nef-dependent intracellular recruitment of pTyr-Lck

**Fig 5:**
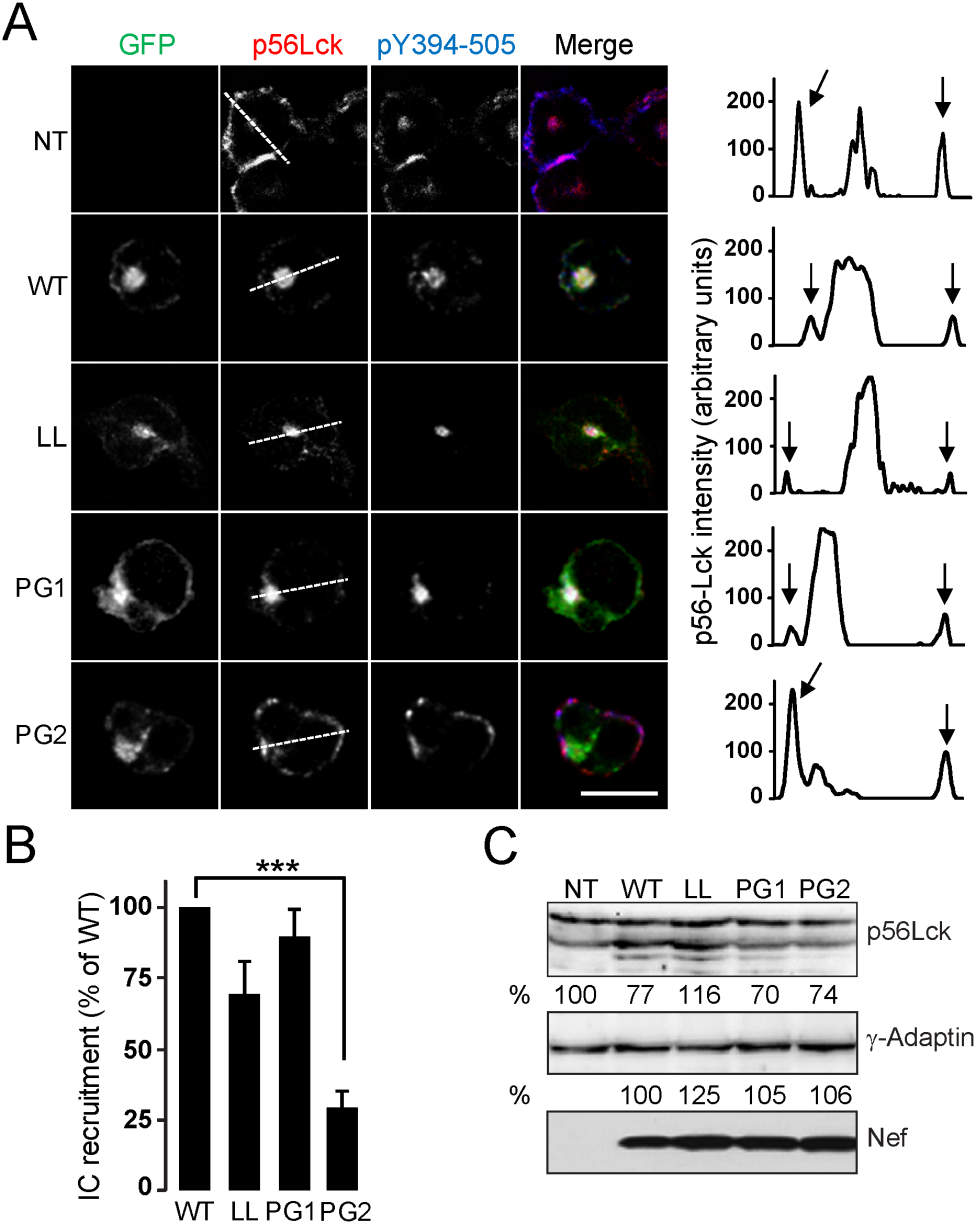
The GPG-motif is required for Nef-induced intracellular recruitment of p56^Lck^. (A-B) The subcellular distribution of endogenous p56^Lck^ and pTyr-Lck was analysed by indirect immunofluorescent staining of non-transfected, NT, or transfected Jurkat cells with the expression plasmids for wild type or the indicated Nef-GPG mutants as GFP-fusion proteins. A) A medial optical section of a representative cell is shown. Densitometry analysis of the distribution of endogenous p56^Lck^ staining along the line in Jurkat cells expressing the indicated Nef-GFP proteins. Arrows indicate the position of the cell periphery (right panels). Scale bar, 10 μm. B) Quantification of the experiment shown in A. The percentage of cells with high levels of intracellular p56^Lck^ was measured as described in Materials and Methods. Histograms represent the cells with predominant intracellular localization of p56^Lck^ compared to control cells, respectively. Values are the arithmetic mean ± SD of at least three independent experiments in which over 100 cells were counted per condition. ***, p< 0.001. C) The expression of endogenous p56^Lck^ and transfected Nef-GFP proteins in Jurkat cells was analysed by western blotting using an anti-p56^Lck^ antibody and an anti-GFP antibody, respectively. **γ**-adaptin was used as loading control.

Nef expression in T-cells promotes the retargeting of p56^Lck^ from the plasma membrane to an intracellular Tf-R-positive endosomal compartment in a PxxP-dependent manner [27,28]. To validate the contribution of the GPG-motif to this Nef activity, Jurkat T-cells were transiently transfected with expression constructs for Nef-PG1/AA or -PG2/AA mutants as GFP-fusions, fixed and immuno-stained with anti- p56^Lck^ antibodies. Immunofluorescent analysis showed that Nef WT and the LL/AA mutant triggered the retargeting of p56^Lck^ from plasma membrane to an intracellular compartment. Staining with an anti-pTyr^505^-Lck antibody that recognizes the dually phosphorylated active pY^505-394^ form [29,37] indicated that the majority of this accumulated kinase was in its dually phosphorylated active form (Fig 5A and 5B).

The PG2/AA mutation significantly impaired the ability of Nef to trigger IC accumulation of p56^Lck^ (Fig 5A, right panels, and Fig 5B for quantitation), whereas the PG1/AA mutation did not have any significant effect. The reported effects of GPG mutants were not related to differences on Nef or p56^Lck^ expression levels (Fig 5C). These results were confirmed in peripheral CD3+CD4+ T-cells (S3 Fig).

Thus, the Nef-GPG motif is required for Nef-induced IC recruitment of phosphorylated p56^Lck^.

### Role of the GPG-motif in Nef’s effects on TCR-induced actin remodelling and surface expression of MHCI

We also investigated the role of the Nef-GPG motif in other Nef-dependent effects. First, we analysed the Nef’s effect on TCR-induced actin remodelling by tracking the formation of circumferential F-actin rich rings after TCR stimulation [38,39,40,41]. TCR engagement triggers rapid remodelling of F-actin at the plasma membrane to facilitate and sustain signal initiation and transmission. When TCR stimulation is provided by stimulatory antibodies coated to a substratum, T-cells adhere, spread and reorganize F-actin into a prominent ring structure at the cell periphery (circumferential F-actin rings). We previously established that Nef does not affect T-cell adhesion to such stimulatory surfaces but impairs their ability to subsequently spread and form circumferential F-actin rings [38,39,40,41]. This effect is strictly dependent on the interaction surface of Nef with the host cell kinase PAK2 and involves phosphorylation events mediated by Nef-associated PAK2 but also the activity of the exocyst complex that is recruited into the Nef complex by PAK2 [42]. Since Nef-PAK2 association strictly depends on the PxxP motif in Nef [43,44,45] we tested whether the GPG-motif is also required for disruption of TCR-induced actin remodelling by Nef.

**Fig 6:**
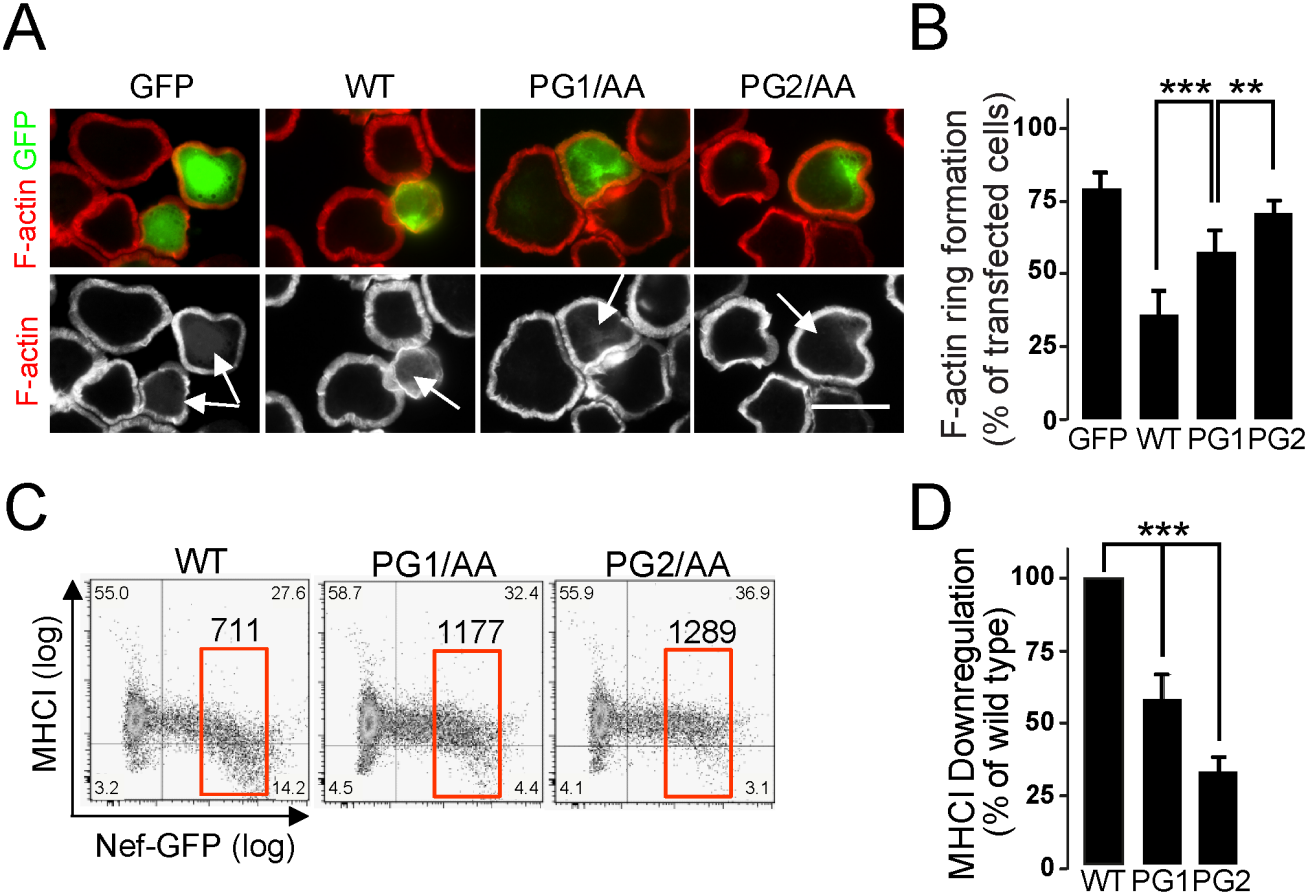
GPG-mutations reduce Nef-dependent inhibition of TCR-induced actin remodelling and Nef-induced down-regulation of MHCI. (A-B) Fluorescent microscopy of Jurkat T-cells transfected with the indicated GFP or Nef-GFP expression plasmids after 5 min incubation on anti-CD3 coated cover glasses and subsequent staining for F-actin. A) Depicted pictures are a representative image at the cell-cover glass level. The upper panel presents a merge of the GFP and F-actin channels, the lower panel shows the F-actin channel only. Arrows indicate transfected cells. Scale bar, 10 μm. B) Quantification of the experiment shown in A. Shown is the percentage of cells exhibiting pronounced F-actin ring formation. Values are the arithmetic mean ± SD of at least three independent experiments in which over 100 cells were counted per condition. ***, p< 0.001. (C-D) CEM cells were transfected with the wild type or the indicated Nef-GPG mutants as GFP-fusion proteins. Nef-induced surface MHCI down-regulation was assessed by flow cytometry in Nef-GFP expressing cells. C) Dot plots and the corresponding MFI are representative of three independent assays. D) The mean fluorescence intensity (MFI) of MHCI (Alexa-555) in the GFP-positive cells was used to calculate activity relative to wild-type Nef. Values are the arithmetic mean ± SD of at least three independent experiments. **, p< 0.01; ***, p< 0.001.

Expectedly, GFP expressing control Jurkat T-cells rapidly spread on surfaces coated with anti-CD3 antibodies and formed circumferential F-actin ring in a manner undistinguishable from non-transfected neighbouring cells (see GFP expressing control cells in Fig 6A). As established previously [30,34,38], Nef potently disrupts cell spreading and formation of F-actin rings, resulting in small rounded cells that remain attached to the surface without undergoing notable actin rearrangements (Fig 6A, note that the Nef.GFP expressing cell is less spread than non-transfected neighbouring cells and does not display prominent F-actin structures at the cell periphery). On unstimulatory surfaces T-cells adhere but do not spread or form circumferential F-actin rings and Nef-expressing cells are undistinguishable from control cells (data not shown and [46]). Upon TCR stimulation, the PG2/AA and PG1/AA mutations significantly reduced the Nef-mediated inhibition of actin dynamics and cell spreading, with effects of the PG2/AA mutation being significantly more pronounced than those of the PG1/AA mutation (Fig 6A). Quantification of over 100 cells each from three independent experiments in which cells were grouped according to their ability to spread and form circumferential F-actin rings (e.g. as the GFP expressing cells in panel A) revealed that the PG1 and PG2 mutants displayed statistically significantly less disruption of F-actin ring formation than wild type Nef but still retain residual activity (Fig 6B).

Second, we investigated the effect of the GPG-motif on Nef-induced downregulation of MHCI. In contrast to CD4, MHCI downregulation by HIV-1 Nef is not related to its interaction with p56^Lck^, but depends on the PxxP motif [11,32,47,48]. Our results show that Nef activity on MHCI downregulation was reduced by 40% and 60% in PG1/AA or PG2/AA-expressing T-cells respectively (Fig 6C and 6D).

Together these results could indicate a weak participation of the GPG-motif in the inhibition of host cell actin dynamics and in MHCI-downregulation by HIV-1 Nef.

### The Nef-GPG motif is required for Nef-dependent optimal infectivity in T-cells

**Fig 7:**
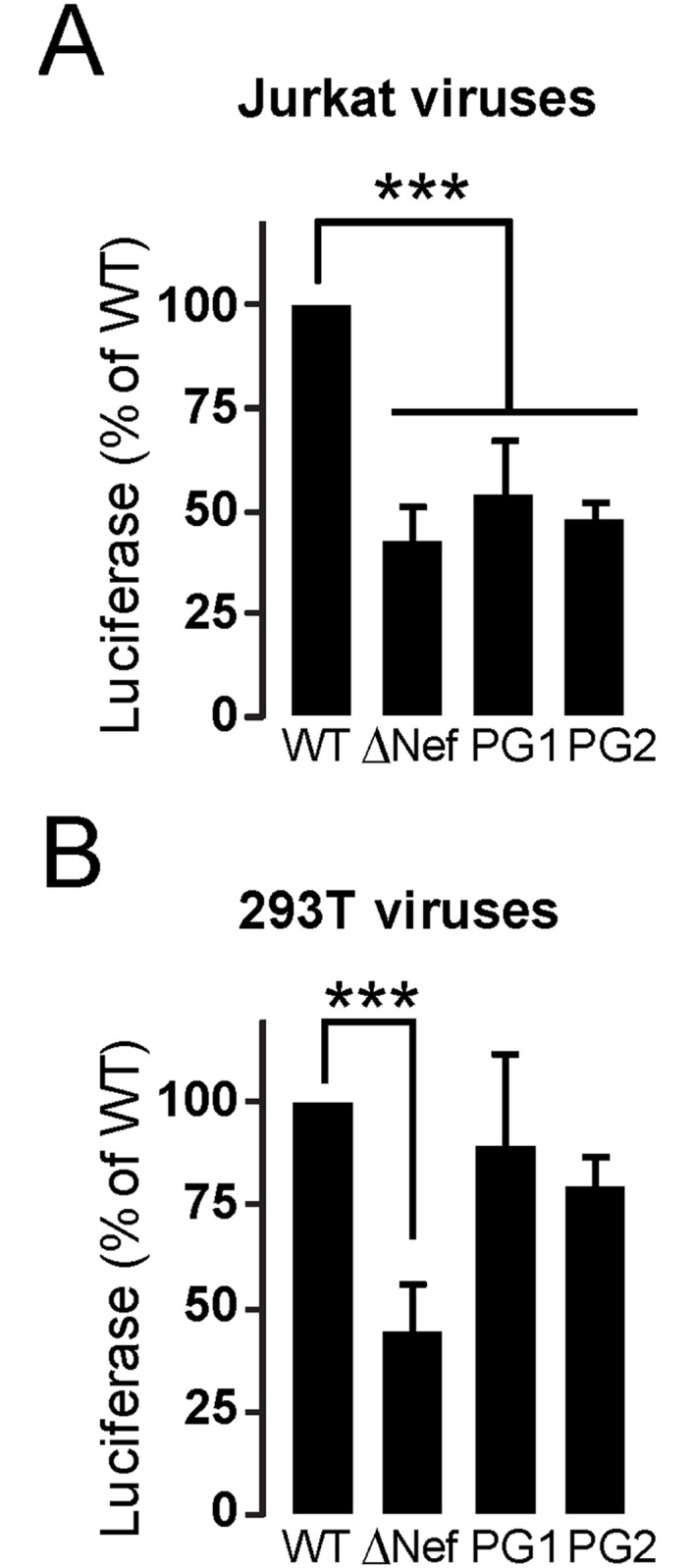
Nef-GPG motif is required for Nef-enhanced infectivity of HIV-1. Relative infectivity of HIV-1expressing Nef-GPG mutations produced in Jurkat (A) or 293T (B) cells. Infectivity was analysed 48h post-infection by Luciferase assays in TZM-bl reporter cells. Results are expressed as the percentage of infectivity relative to that of the wild type viruses normalized to 100%. Values are the mean ± SD of three independent infections, each measured in duplicate. ***, p< 0.001.

Finally, we investigated the contribution of the Nef-GPG motif in Nef-dependent enhancement of viral infectivity. For this purpose wild type, ΔNef, and Nef- PG1/AA or -PG2/AA viruses were produced in Jurkat or 293T cells, and assayed for infectivity at 48h post infection in reporter TZM-bl cells. We found that PG1/AA and PG2/AA viruses produced in Jurkat cells were as deficient as ΔNef viruses, showing similar infectivity levels (Fig 7A).

In contrast, infectivity of viruses produced in 293T cells circumvented the requirement of integral Nef GPG-motif. Indeed, mutant PG1and PG2 viruses produced in these cells were almost as infectious as wild type viruses (Fig 7B and Table 2). These data indicate that the GPG-motif is a new molecular determinant for Nef-dependent optimal infectivity of viruses produced in CD4+ T-cells.

**Table 2:** Summary of Nef-activities related to GPG-domain.

	CD4		Tf-R	AP-1	p56^Lck^ I.C.R	Actin ring	MHCI	Enhanced Viral Infectivity	
	+ Lck	-Lck						Jurkat	293T
WT	++	++	++	++	++	++	+	++	++
QN/AA	+	++	++*	n.d	n.d	+*	-*	n.d	n.d
YT/AA	-	-	-*	n.d	n.d	-*	-*	n.d	n.d
PG1/AA	+	++	++	++	++	+	-	-	++
PG2/AA	-	++	++	++	-	-	-	-	++
VR/AA	-	-	-*	n.d	n.d	-*	-*	-*	-*
YP/AA	-	-	-*	n.d	n.d	-*	-*	n.d	n.d
DW/AA	-	-	-*	+	n.d	++*	-*	n.d	n.d
LL/AA	-	-	-	-	+	++*	+	n.d	n.d

Results are presented from this report unless otherwise indicated. CD4, CD4 downregulation; Tf-R, Tf-R downregulation and uptake of Tfn ligand; AP-1, *in vitro* binding to AP-1 complex by pull down assays; p56^Lck^ ICR, Nef-induced IC recruitment of phosphorylated p56^Lck^ Actin ring, reorganization of actin ring upon CD3-stimulation in T-cells; MHCI, MHCI downregulation; Enhancement of viral infectivity, infectivity of viruses produced by proviral transduction in either Jurkat or HEK293T cells. “++” activity of mutated Nef is >75% of wild type protein: “+” activity is between 25% and 75% of wild type; “-“activity is less than 25% of wild type; n.d. no data.

* not shown.

## Discussion

We report the identification of the HIV-Nef GPG-motif as a novel molecular determinant that contributes to Nef-induced CD4 downregulation, intracellular accumulation of phosphorylated p56^Lck^, and participates in actin-rearrangements upon TCR triggering and in MHCI downregulation. Besides and more importantly, the Nef GPG-motif was also required for Nef-enhanced HIV-1 infectivity of viruses produced in T-cells (for summary, see Table 2). Despite such a general effect on well-known Nef activities, the observed effects by the Nef-PG1 or -PG2 mutations are unlikely to rely on a potential alteration of Nef structure. This assumption is supported by the differential requirement of the Nef-GPG motif on Nef-induced CD4-downregulation in HeLa-CD4 and HeLa-CD4/p56^Lck^ cells. Moreover, our results on Nef/AP-1 interaction and Tf-R trafficking assays, strongly suggest that mutations on the GPG-motif do not promote any significant impact on those motifs required for Nef-induced impairment of endosomal trafficking. Compared to the Nef-D123A mutant, our results may also exclude any negative effect of GPG mutations on the dimerization of HIV-1 Nef. Furthermore, in agreement to a structural report on Nef [49], while the YT, VR and YP residues in the GPG-loop participate in the formation hydrogen bonds to stabilise the core β2, β3 and β4 sheets, the central GPG-motif conforms an hydrophobic domain that is exposed at the protein surface [49]. A potential cooperation between the PxxP domain and the GPG-motif could explain the impairment on F-actin reorganization and Nef-dependent MHCI-downregulation observed in Nef-PG1/AA or -PG2/AA expressing cells.

The fact that the GPG-motif was dispensable for Nef-induced CD4-downregulation in HeLa-CD4 cells but required in T-cells or HeLa-CD4/p56^Lck^ cells, also suggest that the Nef-GPG-motif would be required for early steps of HIV-1 Nef/p56^Lck^ dislodgement previous to CD4-internalization in T-cells, likely through direct or indirect interaction with p56^Lck^. A computational prediction using a motif discovery algorithm identified the GPG-loop in HIV-1 Nef as a potential binding site for p56^Lck^, among other host proteins, in particular its SH2 domain [50]. Since the PxxP motif, the SH3-binding domain in Nef, is intact in Nef-GPG-mutants our ongoing studies are focused to ascertain whether this GPG-motif is involved in the Nef-binding to p56^Lck^-SH2.

We also observed that the GPG-motif was required for Nef-induced IC recruitment of dually phosphorylated pTyr^505/394^, potentially into the RE/TGN as previously illustrated in HIV-1 infected T-cells [29]. This hallmark of Nef proteins [30] mostly correlates with its requirement for CD4-downregulation from plasma membrane in p56^Lck^-expressing cells. Our observations indicate that Nef-dependent IC recruitment of p56^Lck^ already occurs in resting cells, and suggests that HIV-1 Nef could decouple downstream TCR signalling even before TCR-engagement. Since the central GPG-mutants impair Nef’s effects on both IC recruitment of p56^Lck^ and actin-ring formation upon T-cell activation, we expected that Nef-GPG-motif was required for Nef-dependent optimal viral replication and, very likely, for Nef-induced viral spread through cell-to-cell contacts at the virological synapse.

In this line, the breakthrough of this study is the identification of the GPG-motif as a specific molecular element in HIV-1 Nef required for optimal viral infectivity in T-cells. Viruses bearing mutations on the Nef-GPG-motif were as deficient as ΔNef viruses as long as they were produced in T-cells. In contrast, the Nef GPG-motif was dispensable for those viruses produced in non T-cells. Thus it can also be suggested that p56^Lck^, among other cellular factors, could be involved in the participation of the GPG-motif in Nef-dependent optimal viral infectivity. Strikingly, p56^Lck^ interacts with and targets HIV-1 Gag to the plasma membrane to connect with Nef and viral components for assembly and release [51,52,53]. Moreover, Nef inhibits the TCR-dependent T-cell activation in HIV-1 infected cells but remotely activates the Ras-Erk pathway by an intracellular pool of catalytically active p56^Lck^ [27]. Therefore, it can be suggested that Nef could recruit p56^Lck^ to ensure the target of viral proteins to membrane platforms for proper assembly and budding through the exocytic machinery in the infected cell.

Finally and in agreement with the presence of conserved short linear motifs (SLIMs) among unrelated viral species [50,54], we have observed that other proteins like Tax (Human T-lymphotropic virus -1 and 2) and EP402R (African swine fever virus) may encode orthologous GPG-motifs as they bear highly homologous protein cassettes (70% to 78% of identity) to the GPG-loop in HIV-1 Nef, (Martinez-Bonet, unpublished results). This recurrence of the Nef-GPG-motif not only suggests an evolutionary mechanism for the acquisition of functional regions by mimicking host machineries but also could allow the design of wide spectrum therapeutic approaches with activity over different infectious viruses.

## Supporting Information

S1 Fig Mutations on the Nef-dimerization motif abrogate Nef-mediated CD4 down-regulation of surface CD4 on T-cells. A-B) CEM cells were transfected with either wild type or the indicated Nef mutants as GFP-fusion proteins and Nef-induced surface CD4 down-regulation was analysed by two-colour flow cytometry in GFP expressing cells. A) The mean fluorescence intensity (MFI) of CD4 (PE) in the GFP-positive cells was used to calculate the individual activity relative to wild-type Nef. Histograms represent the arithmetic mean ± S.D of at least three independent experiments. ***, p<0.001; n.s. not statistically significant. B) Dot plots and the corresponding MFI are representative of three independent assays. C-D) Peripheral CD3+CD4+ T-cells were transfected with wild type or the indicated Nef mutants as GFP-fusion proteins. C) The mean fluorescence intensity (MFI) of CD4 (PC7) in the GFP-positive cells was used to calculate the Nef activity on surface CD4 expression. Histograms represent the Nef activity of the corresponding GPG mutants relative to wild-type Nef. Values are the arithmetic mean ± SD of at least three independent experiments. ***, p<0.001. D) The expression of either wild type or the indicated Nef mutants as GFP-fusion proteins was analysed by western blotting. Anti **γ**-adaptin was used as loading control staining.
(TIF)

S2 Fig Ectopic expression of p56^Lck^ in HeLa-CD4 cells restores the GPG-requirement for Nef-induced CD4-downregulation. A-B) HeLa-CD4 cells were co-transfected with either the wild type or the indicated Nef-GPG mutants (Nef-GFP) along with an empty (A) or p56^Lck^ -mCherry constructs (B). Nef activity on CD4-downregulation was analysed by indirect immunofluorescent staining. A medial optical section of representative cell is shown. Single Nef-GFP positive cells in (B) are indicated by asterisks, whereas co-transfected cells are indicated by arrows. Areas of co-localization of Nef-GFP and p56^Lck^-mCherry are seen in yellow. Scale bar, 10 μm. C-D) The expression of Nef-GFP (C) or Nef-GFP and p56^Lck^-mCherry (D) was analysed by western blotting. **γ**-adaptin was used as loading control staining.
(TIF)

S3 Fig The PGPG-motif is required for Nef-induced intracellular recruitment of p56^Lck^ in peripheral CD4+ T-cells. (A-B) The subcellular distribution of endogenous p56^Lck^ and pTyr^505^-Lck in CD4+ T-cells was analysed by indirect immunofluorescent staining in peripheral T-cells expressing the wild type or the indicated Nef mutants as GFP fusion proteins. A) A medial optical section of a representative cell is shown. Scale bar, 10 μm. B) Histograms represent the mean percentage ± SD of cells with predominant intracellular localization of p56^Lck^. Values are the arithmetic mean ± SD of at least three independent experiments in which over 100 cells were counted per condition. ***, p<0.001.
(TIF)

S1 Table Primers to generate site-specific mutants in HIV-1 Nef NL4.3. (PDF)
