# Supplementary figures and images for: A Conserved GPG-Motif in the HIV-1 Nef Core Is Required for Principal Nef-Activities
Source: PLoS One. 2015 Dec 23;10(12):e0145239. doi: 10.1371/journal.pone.0145239 (PMC4689412; doi:10.1371/journal.pone.0145239)

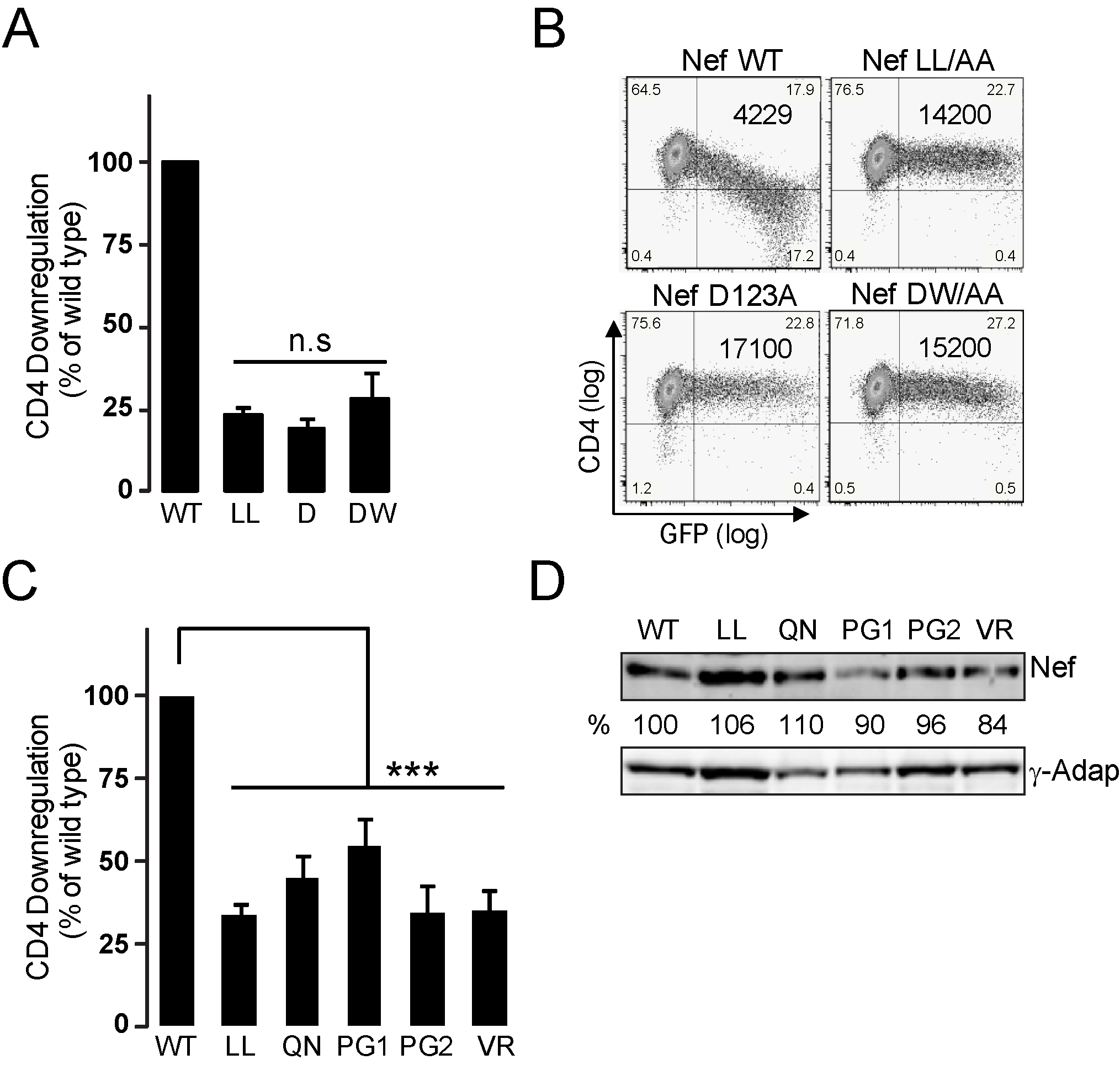

Supplement: S1 Fig — A-B) CEM cells were transfected with either wild type or the indicated Nef mutants as GFP-fusion proteins and Nef-induced surface CD4 down-regulation was analysed by two-colour flow cytometry in GFP expressing cells. A) The mean fluorescence intensity (MFI) of CD4 (PE) in the GFP-positive cells was used to calculate the individual activity relative to wild-type Nef. Histograms represent the arithmetic mean ± S.D of at least three independent experiments. ***, p<0.001; n.s. not statistically significant. B) Dot plots and the corresponding MFI are representative of three independent assays. C-D) Peripheral CD3+CD4+ T-cells were transfected with wild type or the indicated Nef mutants as GFP-fusion proteins. C) The mean fluorescence intensity (MFI) of CD4 (PC7) in the GFP-positive cells was used to calculate the Nef activity on surface CD4 expression. Histograms represent the Nef activity of the corresponding GPG mutants relative to wild-type Nef. Values are the arithmetic mean ± SD of at least three independent experiments. ***, p<0.001. D) The expression of either wild type or the indicated Nef mutants as GFP-fusion proteins was analysed by western blotting. Anti γ-adaptin was used as loading control staining. (TIF) [file pone.0145239.s001.tif]

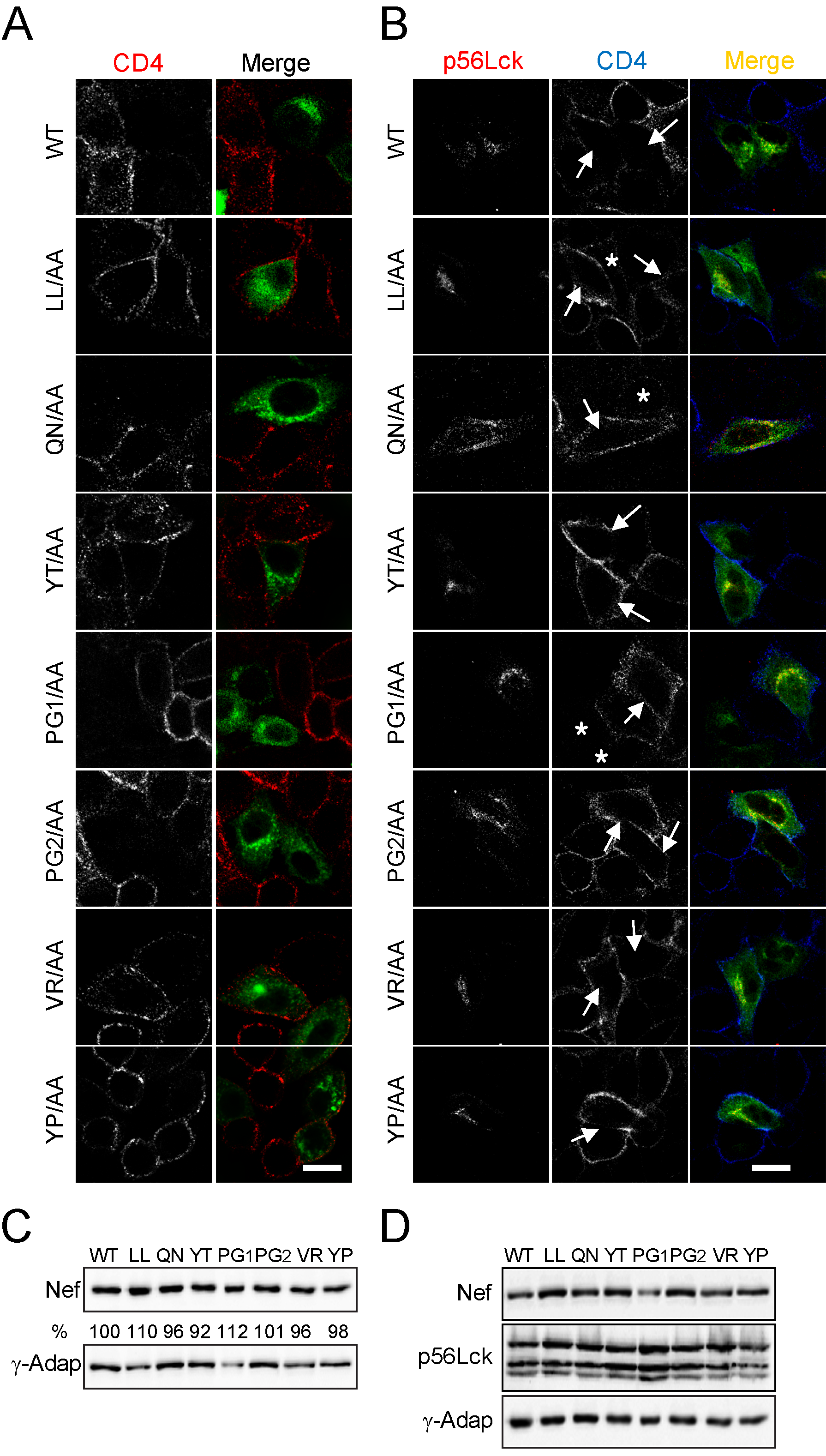

Supplement: S2 Fig — A-B) HeLa-CD4 cells were co-transfected with either the wild type or the indicated Nef-GPG mutants (Nef-GFP) along with an empty (A) or p56Lck -mCherry constructs (B). Nef activity on CD4-downregulation was analysed by indirect immunofluorescent staining. A medial optical section of representative cell is shown. Single Nef-GFP positive cells in (B) are indicated by asterisks, whereas co-transfected cells are indicated by arrows. Areas of co-localization of Nef-GFP and p56Lck-mCherry are seen in yellow. Scale bar, 10 μm. C-D) The expression of Nef-GFP (C) or Nef-GFP and p56Lck-mCherry (D) was analysed by western blotting. γ-adaptin was used as loading control staining. (TIF) [file pone.0145239.s002.tif]

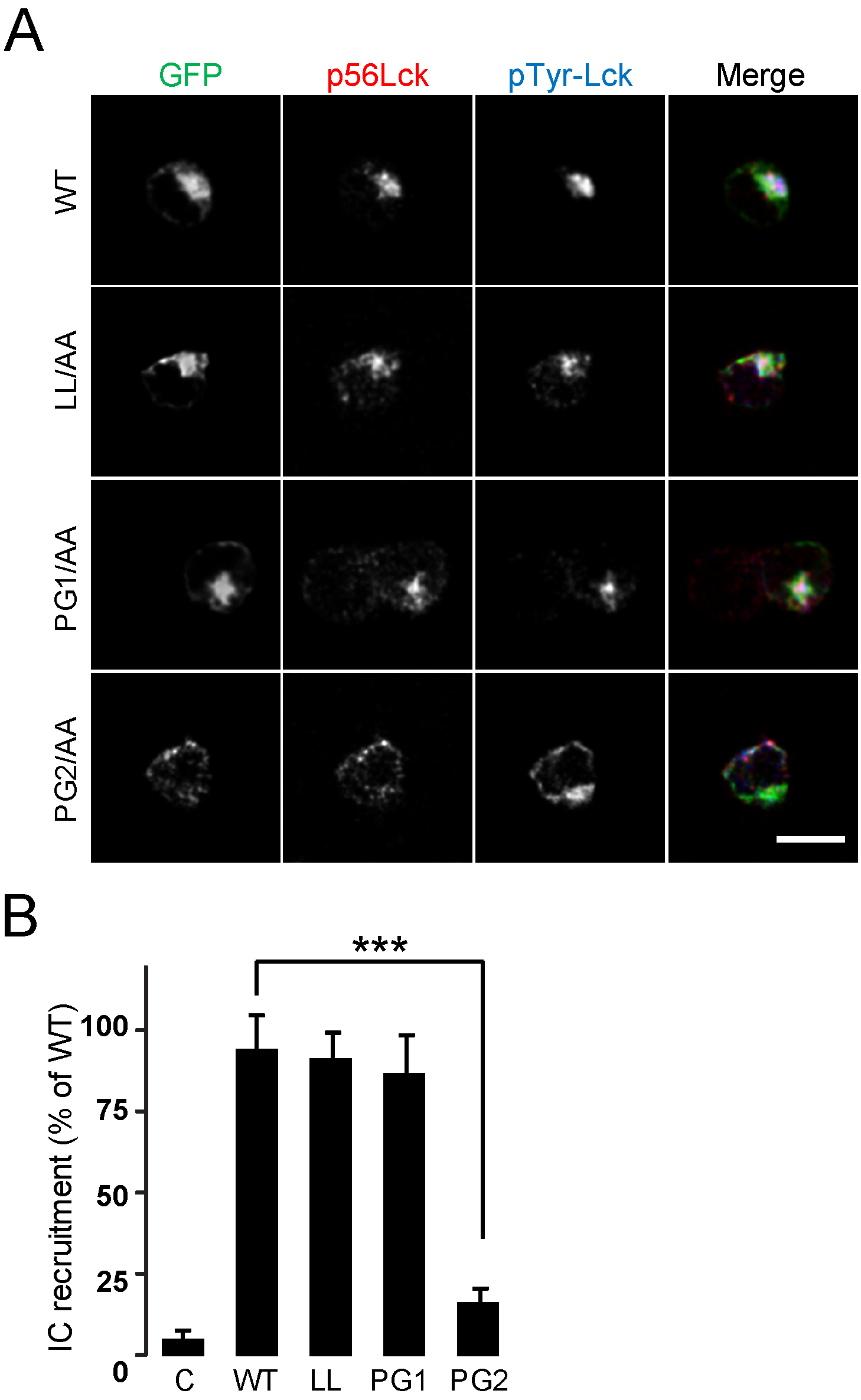

Supplement: S3 Fig — (A-B) The subcellular distribution of endogenous p56Lck and pTyr505-Lck in CD4+ T-cells was analysed by indirect immunofluorescent staining in peripheral T-cells expressing the wild type or the indicated Nef mutants as GFP fusion proteins. A) A medial optical section of a representative cell is shown. Scale bar, 10 μm. B) Histograms represent the mean percentage ± SD of cells with predominant intracellular localization of p56Lck. Values are the arithmetic mean ± SD of at least three independent experiments in which over 100 cells were counted per condition. ***, p<0.001. (TIF) [file pone.0145239.s003.tif]
