# Supplementary material for: A Conserved GPG-Motif in the HIV-1 Nef Core Is Required for Principal Nef-Activities
Source: PLoS One. 2015 Dec 23;10(12):e0145239. doi: 10.1371/journal.pone.0145239 (PMC4689412; doi:10.1371/journal.pone.0145239)
Supplement: S1 Table — (PDF) [file pone.0145239.s004.pdf]

S1 Table

A) Primers to generate Nef dimerization mutants of Nef-GFP, Nef-HA and GST-Nef fusion proteins

Mutation	***FW primer***	***REV primer***
D123/A	5’-GGCTACTTCCCTGCTTGGCAGAACTAC-3’	5’-GTAGTTCTGCCAAGCAGGGAAGTAGCC-3’
D123W124/AA	5’-CAAGGCTACTTCCCTGCTGCGCAGAACTACACACCAGG-3’	5’-CCTGGTGTGTAGTTCTGCGCAGCAGGGAAGTAGCCTTG-3’

B) Primers for site specific mutagenesis on GPG motif of Nef-GFP, Nef-HA and GST-Nef fusion proteins

Mutation	***FW primer***	***REV primer***
Q125N126/AA	5’-CTACTTCCCTGATTGGGCGGCCTACACACCAGGGC-3’	5’-GCCCTGGTGTGTAGGCCGCCCAATCAGGGAAGTAG-3’
Y127T128/AA	5’-CTTCCCTGATTGGCAGAACGCCGCACCAGGGCCAGGGGTCAG -3’	5’-CTGACCCCTGGCCCTGGTGCGGCGTTCTGCCAATCAGGGAAG-3’
P129G130/AA	5’-GGCAGAACTACACAGCAGCGCCAGGGGTCAG-3’	5’-CTGACCCCTGGCGCTGCTGTGTAGTTCTGCC-3’
P131G132/AA	5’-GAACTACACACCAGGGGCAGCGGTCAGATATCC-3’	5’-GGATATCTGACCGCTGCCCCTGGTGTGTAGTTC-3’
V133R134/AA	5’-CTACACACCAGGGCCAGGGGCCGCATATCCACTGACCTTTGGATG-3’	5’-CATCCAAAGGTCAGTGGATATGCGGCCCCTGGCCCTGGTGTGTAG-3’
Y135P136/AA	5’-CCAGGGCCAGGGGTCAGAGCTGCACTGACCTTTGGATGGTG-3’	5’-CACCATCCAAAGGTCAGTGCAGCTCTGACCCCTGGCCCTGG-3’
